# Identification of a clinical signature predictive of differentiation fate of human bone marrow stromal cells

**DOI:** 10.1186/s13287-021-02338-1

**Published:** 2021-05-03

**Authors:** Justyna Magdalena Kowal, Sören Möller, Dalia Ali, Florence Figeac, Torben Barington, Hagen Schmal, Moustapha Kassem

**Affiliations:** 1 Department of Endocrinology, Odense University Hospital, Odense, Denmark; 2 Molecular Endocrinology Unit (KMEB), Institute of Clinical Research, University of Southern Denmark, Odense, Denmark; 3 OPEN - Open Patient data Explorative Network, Odense University Hospital and Department of Clinical Research, University of Southern Denmark, Odense, Denmark; 4 Department of Clinical Immunology, Odense University Hospital, Odense, Denmark; 5 Department of Clinical Research, University of Southern Denmark, Odense, Denmark; 6 Department of Orthopedics and Traumatology, Odense University Hospital, Odense, Denmark; 7 Department of Orthopedics and Trauma Surgery, Medical Center - Albert-Ludwigs-University of Freiburg, Faculty of Medicine, Albert-Ludwigs-University of Freiburg, Hugstetter Straße 55, 79106 Freiburg, Germany; 8 Department of Cellular and Molecular Medicine, Danish Stem Cell Center (DanStem), University of Copenhagen, 2200 Copenhagen, Denmark

**Keywords:** Human bone marrow stromal stem cells, Osteoblastic and adipocytic differentiation, Cell phenotype, Donor characteristics, CD markers

## Abstract

**Background:**

Transplantation of human bone marrow stromal cells (hBMSCs) is a promising therapy for bone regeneration due to their ability to differentiate into bone forming osteoblastic cells. However, transplanted hBMSCs exhibit variable capacity for bone formation resulting in inconsistent clinical outcome. The aim of the study was to identify a set of donor- and cell-related characteristics that detect hBMSCs with optimal osteoblastic differentiation capacity.

**Methods:**

We collected hBMSCs from 58 patients undergoing surgery for bone fracture. Clinical profile of the donors and in vitro characteristics of cultured hBMSCs were included in uni- and multivariable analysis to determine their predictive value for osteoblastic versus adipocytic differentiation capacity assessed by quantification of mineralized matrix and mature adipocyte formation, respectively.

**Results:**

We identified a signature that explained > 50% of variation in osteoblastic differentiation outcome which included the following positive predictors: donor sex (male), absence of osteoporosis diagnosis, intake of vitamin D supplements, higher fraction of CD146+, and alkaline phosphate (ALP+) cells. With the exception of vitamin D and ALP+ cells, these variables were also negative predictors of adipocytic differentiation.

**Conclusions:**

Using a combination of clinical and cellular criteria, it is possible to predict differentiation outcome of hBMSCs. This signature may be helpful in selecting donor cells in clinical trials of bone regeneration.

**Supplementary Information:**

The online version contains supplementary material available at 10.1186/s13287-021-02338-1.

## Background

The clinical efficacy of transplanted human bone marrow stromal cells (hBMSCs) is being tested in an increasing number of clinical trials aiming at enhancing tissue regeneration following injury [1–3]. hBMSCs are easy to isolate from clinical samples and can differentiate into several cell lineages including bone-forming osteoblastic cells which is clinically favorable outcome for bone regeneration [4, 5]. In addition to the differentiation potency, the cells may also be involved in a biological process that supports immunomodulation and tissue regeneration, by secreting paracrine factors [6–8]. Thus, hBMSC transplantation is a promising therapy for bone regeneration for a number of pathologies including non-union and delayed fracture healing or in combination with biomaterials for repairing large bone defects [9–13]. hBMSCs are acknowledged by the Food and Drug Administration Agency (FDA) as suitable adult stem cells for human clinical trials of bone regeneration, as hBMSCs meet the recent recommendations regarding the use of human cell-based products [14].

While the safety of hBMSC transplantation is acceptable [15, 16], the clinical efficacy with respect to bone regeneration varies among trials [3, 13, 17]. A possible explanation is the functional heterogeneity of the transplanted cells and the lack of a set of standardized in vitro criteria for selecting the most appropriate hBMSCs for treatment [3]. Cellular heterogeneity of in vitro cultured hBMSCs are caused by intrinsic factors related to stem cells, i.e., differences in numbers (indicated by colony-forming efficiency or CD (cluster of differentiation) marker expression), proliferation rate, and factors related to their differentiation capacity, i.e., the ability of the cells to differentiate into bone-forming osteoblastic cells or cells of alternative lineages such as adipocytic cells, which is considered an unwanted outcome when developing therapies for bone regeneration [18–22]. Furthermore, cultured hBMSCs may exhibit variations in their biological characteristics caused by extrinsic factors, i.e., donor age, sex, or the presence of metabolic bone diseases [23–25]. Thus, the clinical use of hBMSCs requires determining the relative contribution of donor-related phenotype and intrinsic cellular characteristics, on osteoblast differentiation outcome, with the aim of selecting the most optimal hBMSC product for clinical applications.

To address these points, we conducted a prospective study, where we obtained bone marrow samples from a clinical cohort undergoing surgery for bone fracture. We determined the biological characteristics of the hBMSCs and correlated these parameters with the clinical phenotype of each individual patient including health profile and lifestyle factors. Using univariable and multivariable analysis, we identified a set of variables predictive for the ability of the cultured hBMSCs to differentiate into bone-forming osteoblastic cells.

## Methods

### Donors and materials

The bone marrow was aspirated from the lower extremities of 58 adult donors undergoing surgery at the Department of Orthopedic Surgery and Traumatology, Odense University Hospital, Odense, Denmark. Due to the lack of preliminary data to calculate the sample size and to the exploratory character of the study with unknown variables, we collected the highest possible number of specimens for a duration of 1 year 2016/2017. Collected samples were categorized as “waste material” and the procedure did not pose any additional risk for the patients. All subjects received oral and written information and signed a consent form. Information regarding the health status of each donor was obtained from the patient journal provided by the Danish Healthcare System and during the interview with the medical professional. The project was approved by the Scientific Ethical Committee of the region of Southern Denmark (project ID: S-20160084). Parts of the data from the manuscript have been correlated with cell morphology data in a separate study published by our group [22].

### Cell isolation and culture

Bone marrow aspirates (5–10 ml) were collected into ethylenediaminetetraacetic acid (EDTA)-coated tubes. hBMSCs were isolated from the mononuclear cell fraction following gradient centrifugation on Lymphoprep®, followed by plastic adherence [25]. The cells were cultured in minimum essential medium (MEM medium) supplemented with 10% fetal bovine serum (FBS) and 1% penicillin/streptomycin (P/S) at 37^o^C in humidified 5% CO_2_ incubator. After a week, when the first cells adhered to the plastic surfaces, the media were switched to MEM media including 10% FBS, 1% P/S, 1% GlutaMAX, 1% sodium pyruvate, and 1% non-essential amino acids (S-MEM growing medium). The same batch of FBS was used throughout the study. At 80% confluence, the cells were trypsinized and used for subsequent analysis.

### Colony-forming unit-fibroblast (CFU-f) assay

CFU-f assay was performed in triplicates. The freshly isolated cells were counted under an optical microscope using a hemocytometer and plated at a density of 1 million cells (passage 0) into each of three 22.1cm^2^ Petri dishes (TPP, 93060) and cultured for 17 days under standard culture conditions. The colonies were visualized by crystal violet staining.

### Cell proliferation

Cell proliferation capacity was performed in triplicates at the first cell passage. The cells were counted under an optical microscope using a hemocytometer and subsequently seeded (1000 cells/well) in a 6-well plate (TPP, 92006) in triplicates and cultured under standard conditions. At day 1, 3, 6, 9, 12, and 15, the cells were trypsinized and counted in a hemocytometer, and the proliferation capacity of the cells was measured as the area under the curve (AUC). The population doubling time (PDT) in hours between days 1 and 6 was calculated using the following formula: PDT=120hours*log(2)/(log(Ncellsday6/Ncellsday1)).

### Flow cytometry

hBMSCs after ex vivo expansion to passage 2 were trypsinized and washed with phosphate-buffered saline (PBS) (without Ca^2+^ and Mg^2+^) containing FBS (2%). The cells were incubated with primary fluorophore-conjugated antibodies as follows: CD146-PE, CD271-FITC, ALPL-APC, PDGFRα-PE, CD34-PE, PDPN-APC, CD164-PE, CD362-PE, and CXCR4-PE for 25 min at 4^o^C. After the incubation, cells were analyzed using BD LSR II Flow Cytometer (BD FACSDiva). The data were analyzed with Kaluza Flow Cytometry Analysis Software Version 1.3 (Beckman Coulter).

### In vitro cell differentiation

#### Osteoblastic differentiation

hBMSCs from passage 1 were seeded (20.000 cells/cm^2^), and after 24h, the media were replaced with osteoblastic induction media supplemented with: 10% FBS, 1% P/S, 5mM β-glycerophosphate, 10nM dexamethasone, 50μg/ml vitamin C, and 10nM vitamin D_3_. The media were changed every 2–3 days. After 14 days, the osteoblastic differentiation was assessed. The osteoblastic differentiation was performed in duplicates.

##### Alizarin red staining

For visualization of mineralized matrix formation, alizarin red staining was performed. The cells were washed with PBS and fixed with 70% ice-cold ethanol at −20^o^C for 1h, washed with H_2_O, and incubated with alizarin red (pH=4.2) for 10 min with rotation at room temperature (RT). The stained cells were scanned and the potency of the cells to form mineralized matrix was quantified as the intensity of alizarin red using ImageJ software and expressed in arbitrary units (AU).

##### Alkaline phosphatase (ALP) activity

The cells were washed with tris-buffered saline (pH 9), fixed with formaldehyde-ethanol for 30 s at RT, and incubated with p-nitrophenyl phosphate (1mg/ml) in 50mM NaHCO_3_ and 1 mM MgCl_2_, pH 9.6 at 37^o^C. After 20 min of incubation, 3M NaOH was added to stop the reaction. Absorbance was measured at 405 nm, and ALP activity values were corrected for a number of hBMSCs in each well. The cell number was determined based on cell viability and determined by incubating the cells with CellTiter-Blue for 1h at 37^o^C. The fluorescent intensity (560ex/590em) was measured in FLUOstar Omega plate reader. In independent experiments from our laboratory, cell viability measurements showed excellent correlation with cell numbers determined by manual counting of the cells.

##### ALP staining

The cells were fixed in acetone-citrate buffer (5:1) for 5 min at RT. The cells were then incubated with naphthol/fast red solution for 1h at RT.

#### Gene expression of osteoblastic markers using quantitative real-time PCR (qRT-PCR)

The total RNA was isolated from the cells using TRIzol reagent and following the protocol provided by the manufacturer. The reverse transcription was performed using a High Capacity cDNA Reverse Transcription Kit. Quantitative real-time PCR was performed with an Applied Biosystems 7500 Real-Time PCR System using Fast SYBR Green Master Mix with primers of the following genes: Collagen 1A (*COL1A*), bone sialoprotein (*BSP*), and osteocalcin (*OCN*). The sequences of the primers are included in Supplementary Table 1. Gene expression data were normalized to β-actin housekeeping gene and expressed as delta-delta Ct values.

##### Adipocytic differentiation

hBMSCs from passage 1 were seeded (30.000 cells/cm^2^). At near full confluency, the media was replaced with adipocytic induction media containing Dulbecco’s modified Eagle’s medium (DMEM) supplemented with 10% FBS, 1% P/S, 5% horse serum, 1μM rosiglitazone (BRL), 3μg/ml insulin, 100nM dexamethasone, and 225μM 3-isobutyl-1-methylxanthine (IBMX) and changed every 2–3 days. After 14 days, adipocytic differentiation efficiency was determined. The adipocytic differentiation was performed in duplicates.

##### Oil Red O staining

The formation of mature adipocytes containing lipid droplets was visualized using Oil Red O staining. The cells were fixed with 4% paraformaldehyde (PFA) for 10min at RT, washed with 3% isopropanol, and incubated with filtered Oil Red O solution (25mg of Oil Red O in 5ml of 100% isopropanol and 3.35 ml H_2_O). Photomicrographs of the cells were captured using an Olympus optical microscope (×10 magnification objective) and quantified as the area of lipid droplets (average of 6 images per sample) using ImageJ software and expressed in arbitrary units (AU).

#### Reagents

LymphoprepTM (StemCell Technologies, 1114545), minimum essential media (MEM, Gibco, 31095-029), Dulbelcco’s modified Eagle’s medium (DMEM, Gibco, 31966), fetal bovine serum (TherFisher, 10270106, lot:42F0266K), GlutaMAXTM (Gibco, 35050-038), non-essential amino acids (MEM NEAA, Gibco, 11140-035), Trypsin-EDTA (Invitrogen, 25300062), β-glycerophosphate (Calbiochem, 35675), dexamethasone (Sigma, D4902), vitamin C (L-Ascorbic Acid Phosphate Magnesium Salt n-Hydrate, Wako, 013-12061), vitamin D3 (1α,25-Dihydroxyvitamin D_3_ a kind gift from Leo Pharma), p-nitrophenyl phosphate (Sigma, 71768), Alizarin Red (Sigma, A5533), Oil Red O (Sigma, O0625), horse serum (Sigma, H1270), rosiglitazone (BRL, Cayman Chemical, 71740), insulin (Sigma, I9278), 3-isobutyl-1-methylxanthine (IBMX, Sigma, I5879), Naphthol AS-TR phosphate disodium salt (Sigma, N6125), Fast Red TR Salt hemi(zinc chloride) salt (Sigma, F8764), anti-CD146 (Beckman Coulter, A07483), anti-CD271 (BioLegend, 345104), anti-ALPL (R&D Systems, FAB1448A), anti-CD164 (Miltenyi Biotec; 130-108-069), anti-PDGFRα (BD Biosciences, 556002), anti-CD34 (BD Biosciences; 555822), anti-PDPN (Miltenyi Biotec; 130-106-955), anti-CXCR4 (R&D Systems, FAB170P), anti-CD362 (Miltenyi Biotec; 130-107-480), CellTiter-Blue cells viability assay reagent (Promega, G8081), TRIzol (Invitrogen, 15596018), High-Capacity cDNA Reverse Transcription Kit (Applied Biosystems™, 4368813), and Fast SYBR™ Green Master Mix (Applied Biosystems™, 4385614).

### Data analysis

Analyses were performed using GraphPad Prism 7.1 and Stata 15.1 software. Data are shown as mean ± SD, unless otherwise stated. Statistical significance was considered when *p*≤0.05. For age, we divided the individual into young (18–45 years), middle-aged (46–65 years), and elderly (>65 years), based on the epidemiological studies of changes of bone mass with aging. BMI was divided into lean (<25), overweight (25–29.9), and obese (>30), similar to the World Health Organization criteria. The normal distribution of all investigated cell and donor-related variables was tested by performing D’Agostino & Pearson normality test. Correlations between variables were investigated using the Pearson or Spearman two-tailed correlation test (*r*_*s*_= correlation coefficient) depending on normality test results. The differences in the distribution of donor population were analyzed with Fisher’s exact test.

#### Multivariable analysis

We performed analysis for the desired outcome of osteoblastic differentiation based on the alizarin red staining and the undesired outcome of adipocytic differentiation based on the oil red O staining. We applied linear regression with stepwise backward selection collectively on clinical parameters (sex, age, body mass index (BMI), presence of osteoporosis, hypertension, diabetes, intake of vitamin D and calcium supplementation, current cigarette smoking, and alcohol consumption) and in vitro cell characteristics (expression of CD146, ALP, CD271, PDGFRα, CD362, CXCR4, CD14, CD34 markers, cell proliferation, number of ALP+ colonies, total number of colonies, and ALP activity at baseline and after osteogenic induction) with a cut-off for exclusion of *p* = 0.10 and reporting coefficients with 95% confidence intervals, standardized to 1 SD change in case of numerical predictors, and *R*^2^ of the resulting regression models. Donors with incomplete information regarding clinical or cellular parameters were excluded from the multivariable analysis. To avoid possible sources of bias in a multivariable model, the data were analyzed by a researcher who was not familiar with the findings of the univariable analysis and did not have an expertise in the cell biology field. This allowed for an objective examination of the predictive value of analyzed variables. For artwork, Servier Medical Art by Servier under a Creative Commons 3.0 license was used.

## Results

### Clinical characteristics of study participants

The clinical phenotype of the 58 participants of the study is shown in Table 1. The studied cohort included both males and females of a wide range of ages (18–97 years) and BMI (17.5–44). hBMSCs were isolated from bone marrow aspirates collected from the femur, tibia, or pelvis. Table 1 also shows the distribution of the following factors within the group: cigarette smoking, alcohol consumption, intake of vitamin D and calcium supplements, clinical biochemistry data, and the presence of the following diseases: osteoporosis, hypertension, and type 2 diabetes. The database also includes information about fracture age, indicating the time of the bone marrow collection from operated fractures, including acute and non-union fractures. In addition, we classified the participants according to the use of medication into two categories: current regular intake of more or less than 3 drugs that included statins, pantoprazole, paracetamol, morphine, or non-steroidal anti-inflammatory drugs.

**Table 1:** Clinical characteristics of the donors

**Variable**	**Distribution (*n*** **of donors)**
Males/females	26/32
Age (18–45 years/46–65 years/ >65 years)	19/16/25
BMI (<25/25–29.9/≥30)	24/16/18
Bone marrow aspiration site (femur/tibia/pelvis)	25/27/6
Cigarette smoking (currently smoking/non-smoking)	21/37
Alcohol consumption ≥8g/day (yes/no)	37/21
Osteoporosis (yes/no)	22/36
Diabetes type 2 (yes/no)	10/48
hypertension (yes/no)	21/37
Number of medications (<3/3≤)	45/23
Vitamin D supplementation (yes/no)	20/46
Calcium supplementation (yes/no)	20/46
**Variable**	**Average (range)**
Weight (kg)	79.5 (44–130)
Height (cm)	170.4 (152–192)
Haemoglobin (mmol/l)	7.7 (5–9.6)
Leucocytes (cells/μl)	9.7 (5–17.6)
C-reactive protein - CRP (mg/l)	34.9 (0.6–244)
Fracture age (days)	79 (1–1095)

### Cultured populations fulfilled minimal hBMSC criteria

Individual hBMSC isolates (strains), each derived from one single individual, were characterized in vitro (Table 2). Cultured hBMSCs formed colonies (i.e., CFU-f), and many of these expressed alkaline phosphatase (mean ± SD, 37.5 ± 24.5%). The proliferative potency of the hBMSCs calculated as PDT was 73.3 ± 34 hours for all donors. We used area-under-the-curve (AUC) as a summary variable of cell proliferation for each donor. In preliminary experiments, we examined hBMSC surface markers recommended by the International Society for Cellular Therapy (ISCT, [26]). We tested cells from 15 consecutive donors and found that ≥ 98% of cell populations demonstrated positive expression of CD44, CD90, CD105, and CD73 and with minimal inter-individual variations [22]. These data confirmed that the cells in our study fulfilled the criteria of hBMSCs. However, these standard CD markers were not suitable as predictive markers due to the absence of inter-individual variations and they were not determined in the remaining cohort.

**Table 2:** In vitro characteristics of hBMSC strains

Variable	Mean ± SD	Number of cell strains tested
Number of ALP positive CFU-f colonies per 1 million plated cells	9 ± 13	48
Number of all CFU-f colonies per 1 million plated cells	22 ± 24	50
ALP activity (baseline)	0.3 ± 0.36	56
ALP activity (induced)	2.3 ± 2.1	58
CD14+ cells (%)	6.8 ± 7.4	58
CD34+ cells (%)	13.2 ± 18	58
CD146+ cells (%)	61.7 ± 30.3	58
ALP+ cells (%)	27.1 ± 17.7	58
CD271+ cells (%)	26 ± 24.8	58
PDGFRα+ cells (%)	37.6 ± 28.1	58
CD362+ cells (%)	30.3 ± 32	56
CXCR4+ cells (%)	12.2 ± 21.2	55
CD164+ cells (%)	96.4 ± 7.1	33
PDPN+ cells (%)	11.2 ± 8.7	32

### Classification of study participants based on hBMSC differentiation outcome

**Fig. 1:**
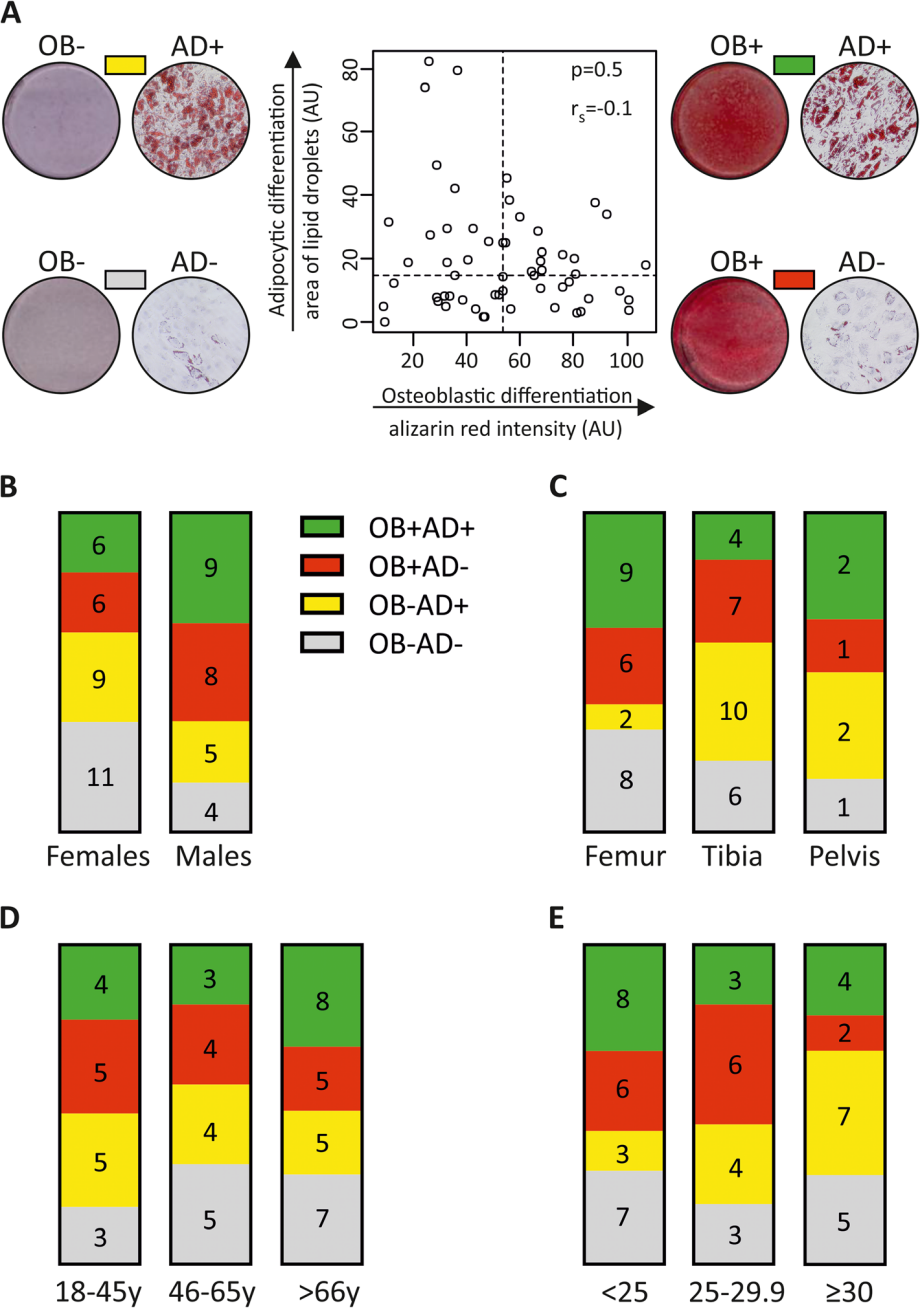
Distribution hBMSC population according to osteoblastic and adipocytic differentiation outcome. Human bone marrow stromal cells (hBMSCs) were obtained from 58 donors undergoing surgery for bone fractures and were induced into osteoblasts (OB) or adipocytes (AD). The differentiation outcome was demonstrated by the ability of the cells to form mineralized matrix stained with Alizarin red (OB) or lipid-filled mature adipocytes stained with Oil red O (AD). **a** The median values of Alizarin red intensity and lipid droplets area were chosen to classify hBMSC differentiation outcome of all donors, into: (I) donors with low osteoblastic and high adipocytic (OB-AD+), (II) donors with high osteoblastic and adipocytic (OB+AD+), (III) donors with high osteoblastic and low adipocytic (OB+AD-), and (IV) donors with low osteoblastic and adipocytic differentiation (OB-AD-). The representative photomicrographs illustrate matrix mineralization and lipid droplet accumulation. The correlation between the osteoblastic and adipogenic differentiation potency of hBMSCs was analyzed using Spearman correlation test. Column graphs illustrate the distribution of donor characteristics with respect to differentiation outcome: **b** donor sex, **c** site of bone marrow aspiration, **d** donor age, and **e** donor BMI. None of the donor-related factors was significantly linked with OB or AD differentiation (Fisher’s exact test)

The aim of the study was to use the differentiation capacity of individual hBMSC strains as a clinically relevant outcome. We therefore quantified the ability of the hBMSCs to differentiate into osteoblastic cells (OB), based on mineralized matrix formation or to differentiate into the alternative undesired outcome; mature adipocytes (AD), based on measuring the area occupied by lipid droplets. As shown in Fig. 1a, we categorized the hBMSC strains (each derived from an individual donor) according to the ability to differentiate into OB or AD, into four groups: good at OB and AD differentiation (OB+AD+), good at OB but poor at AD differentiation (OB+AD−), poor at OB and good at AD differentiation (OB−AD+), poor at differentiation into OB and AD (OB−AD−). This classification was based on whether the quantitative differentiation outcome was above or below the median value of the whole cohort (Fig. 1a and the representative photomicrographs). Using Fisher’s exact test, we did not detect significant associations between the basic donor characteristics (sex of the donor, bone marrow sampling site, donor age, and BMI) and OB or AD differentiation (Fig. 1b–e).

In addition, we determined gene expression of a number of osteoblast lineage genes: *BSP* and *COL1* (Supplementary Figures 1, 2) at day 14, which is a typical endpoint of OB differentiation. We detected a significant correlation (Supplementary Figure 2) between *BSP* expression which is a late marker of OB differentiation and alizarin red staining (Supplementary Figure 2A). Also, *BSP* expression was higher in the cell strains with the highest OB differentiation (Supplementary Figure 1B). For *COL1A* and *OCN* expression (both are early markers OB differentiation) [27], the correlation with Alizarin red staining was not significant (Supplementary Figure 2B-C) as well their ability to identify cell strains with the highest OB differentiation outcome (Supplementary Figure 1B-C). When we combined the 3 gene markers (Supplementary Figure 2D), the correlation with Alizarin red staining improved but was not statistically significant.

### Univariable correlations between donor characteristics and in vitro differentiation outcome of cultured hBMSCs

**Fig. 2:**
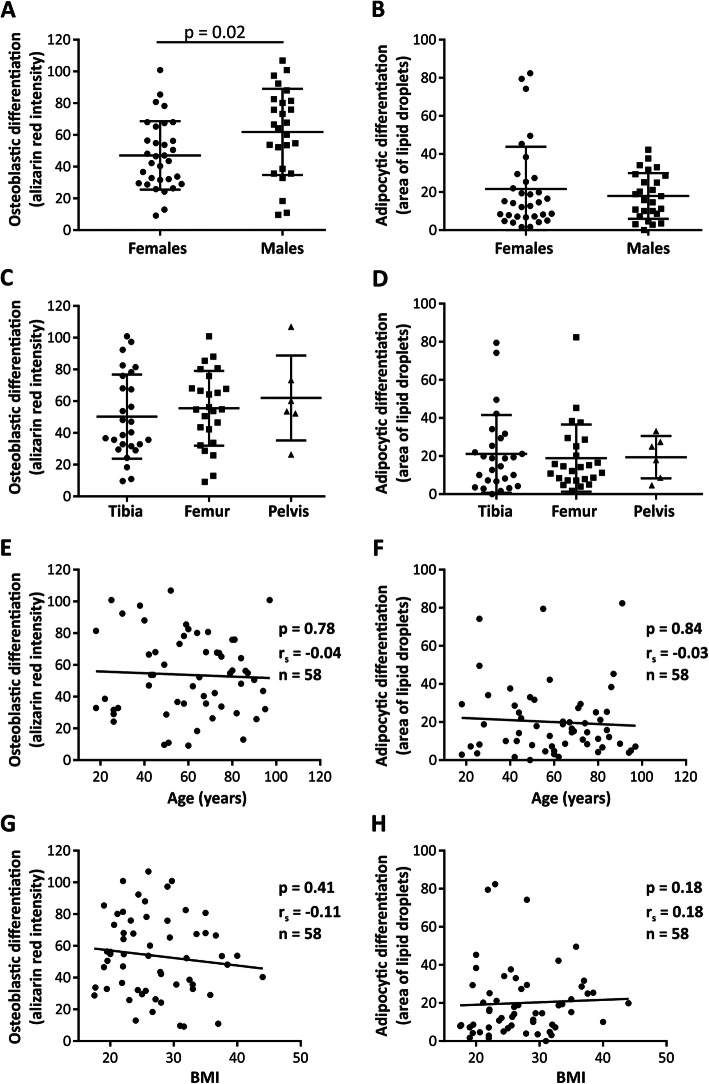
Relationships between donor characteristics and differentiation outcome of hBMSCs. **a**, **b** The impact of donor sex (Student *t* test; *p < 0.05), **c**, **d** the site of bone marrow origin (ANOVA test), **e**, **g** donor age, and **g, h** BMI (Spearman correlation), on osteoblastic and adipocytic differentiation outcome, respectively. Each dot represents the average value of cultured cells from a single donor and *r*_s_ = Spearman’s rank correlation coefficient and *n*= number of tested cell strains, each derived from a single donor

To identify the variables determining the differentiation outcome of hBMSCs, we performed univariable analysis (Fig. 2). We observed increased OB differentiation outcomes among male hBMSCs compared to female hBMSCs (Fig. 2a, *p* = 0.02, *n* = 26 and 32, respectively). In contrast, we did not find any impact of donor sex on adipocytic differentiation (Fig. 2b, *p* = 0.87). The site of bone marrow aspiration (Fig. 2c, d), donor age (Fig. 2e, f), or BMI (Fig. 2g, h) were not associated with a significant impact on OB− or AD differentiation. In addition, we examined the correlation between differentiation outcome and the clinical variables: clinical biochemistry data, anthropometric data, concurrent diseases, current medications, life-style factors, and fracture age. The results provided for all participants (and divided between male and female subjects) are presented in supplementary Tables 2 and 3. Among all analyzed parameters, donor weight was positively correlated with AD differentiation of hBMSCs in female donors (*p* = 0.03), while in male donors, fracture age (<7 days) had a positive impact on OB differentiation of hBMSCs compared to cells from donors with higher fracture age (>7 days) (*p* = 0.04, *n* = 15 and 9, respectively).

### Univariable correlation between in vitro features and differentiation outcome of hBMSCs

**Fig. 3:**
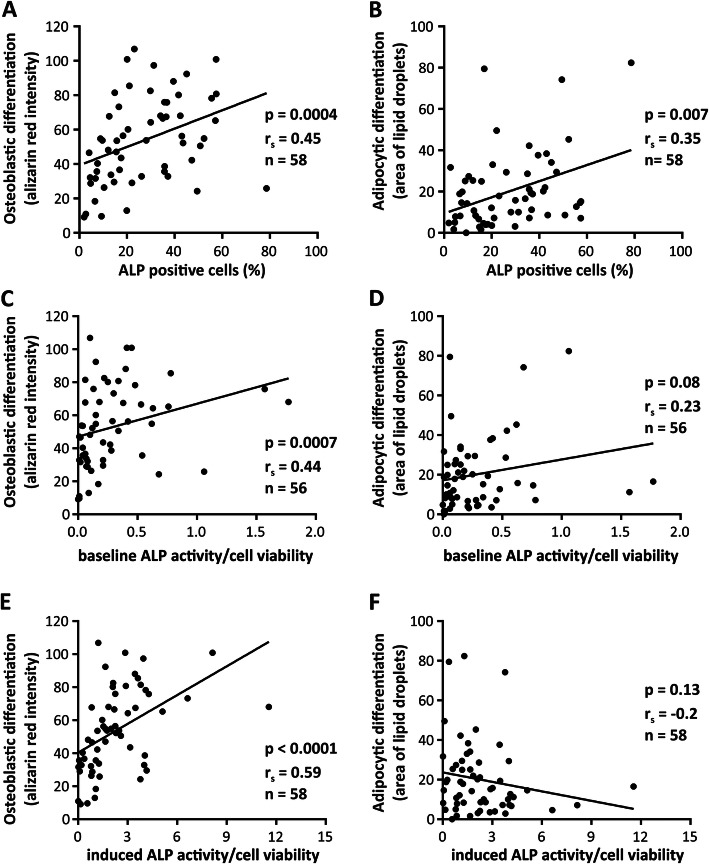
Correlation of alkaline phosphatase (ALP) with differentiation outcome of hBMSCs. **a**, **b** The correlation of percentage of ALP+ cells, **c, d** ALP activity measured at baseline, **e**, **f** ALP activity measured at day 7 of osteoblastic differentiation, with the final osteoblastic and adipocytic differentiation outcome, respectively. Analyzes were performed using Spearman correlation test. Each dot represents the average value of cultured cells from a single donor, *r*_s_ = Spearman’s rank correlation coefficient, and *n* = number of tested cell strains, each derived from a single donor

We examined if the standard characteristics of cultured hBMSCs could predict their differentiation outcome. Both OB and AD differentiation were positively correlated with the percentage of ALP+ cells (Fig. 3a, b, *p* = 0.0004 and *p* = 0.007, *n* = 58). On the other hand, the enzymatic activity of ALP measured at baseline, positively correlated only with OB differentiation (Fig. 3c, *p* = 0.0007, *n* = 56), and not with AD differentiation (Fig. 3d, *p* = 0.08, *n* = 56). Similarly, ALP activity measured at day 7 following culturing the cells in OB induction medium had a strong positive correlation with OB differentiation (*p* < 0.0001, *n* = 58) but not with AD differentiation (Fig. 3e, f).

We did not detect a significant correlation between the proliferative capacity of the cells (Supplementary figure 3A-B), the number of total CFU-f colonies, or ALP+CFU-f colonies (Supplementary figure 4A-E) and OB or AD differentiation. Similarly, we did not observe any significant correlations when the studied cohort was analyzed according to donor sex (Supplementary Table 4).

### Univariable correlations between expression of cell surface marker and differentiation outcome of hBMSCs

**Fig. 4:**
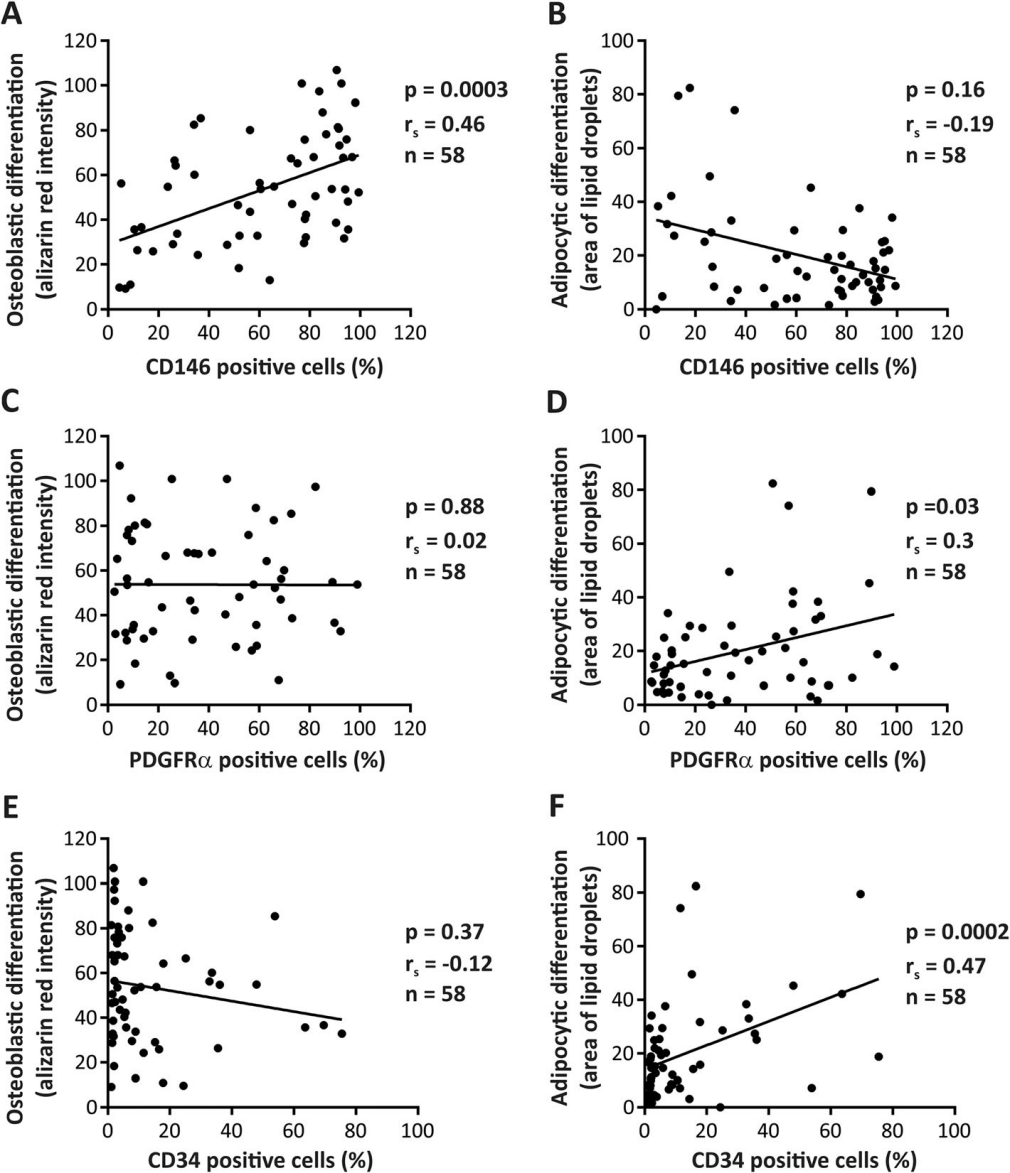
Correlation of cell surface marker expression with differentiation outcome of hBMSCs. **a**, **b** The correlation between the percentage of CD146+ cells **c**, **d** PDGFRα+ cells, **e**, **f** CD34+ cells, and osteoblastic and adipocytic differentiation outcome. Analyzes were performed using Spearman correlation test. Each dot represents the average value of cultured cells from a single donor, *r*_s_ = Spearman’s rank correlation coefficient, and *n* = number of tested cell strains, each derived from a single donor

The standard CD markers (CD44, CD73, CD90, CD105) recommended by ISCT [26] were homogenously expressed (>98% positive) [22] in all tested cell strains. Regarding the negative CD markers recommended by ISCT, CD45 was homogenously expressed (<95% negative). These markers were therefore not useful in explaining inter-individual variations in OB differentiation outcome. We therefore chose to focus on the negative markers CD14 and CD34 which varied among the samples and a number of novel surface markers of hBMSCs [28–30] that were heterogeneously expressed among different cell strains. As shown in Fig. 4, univariable analysis demonstrated that the number of CD146+ cells positively correlated with the OB differentiation (Fig. 4a, *p* = 0.0003), whereas the number of PDGFRα+ and CD34+ cells exhibited positive correlations with AD differentiation (Fig. 4d, f, *p* = 0.03, *p* = 0.0002, respectively). We also tested the relationship between the expression of CD271 [31, 32], CD362 (a marker associated with immunomodulation of hBMSCs [33]), CXCR4 (receptor associated with homing properties of hBMSCs [34, 35]), CD164 and podoplanin (PDPN) membrane markers that have recently been reported to define multipotent human skeletal stem cells [36], and hBMSC differentiation outcome. Although, these markers were heterogeneously expressed in hBMSC populations, we did not observe any significant correlations between the number of cells expressing these markers and the differentiation capacity of the cells (Table 2 and Supplementary Figure 5).

### Multivariable analysis

We performed multivariable analysis using a stepwise regression model with OB or AD differentiation as the main outcome. As we detected that there was a difference between males and females in our study, we performed a post hoc analysis of age-sex interaction and this analysis demonstrated the absence of significant effects on OB and AD differentiations. These findings indicate that there is no interaction between age and sex of the hBMSC donors in predicting the AD/OB outcome and support the findings that the sex effect was not confounded by donor age. To fully investigate the importance of all variables collected in the cohort, we further performed multivariable analysis including all donor- and cell-related variables to identify a group of factors that are predictive for in vitro osteoblast or adipocyte formation.

The list of factors that was selected as highly contributing (significance cut-off *p* < 0.1) to OB or AD differentiation is shown in Table 3. We identified six variables that were predictive for OB differentiation: sex of the donor (*p* = 0.001), presence of osteoporosis (*p* = 0.004), intake of vitamin D supplements (*p* = 0.003), fraction of CD146+ cells (*p* = 0.004), fraction of ALP+ cells (*p* = 0.005), and CD14+ cells (*p* = 0.089).

**Table 3:** List of significant (*p* < 0.1) variables selected by multivariable analysis as predictive for osteoblastic and adipocytic differentiation outcome of cultured human bone marrow stromal cells (hBMSCs)

Variable	***N*** of samples	Standardized regression coefficient (95% CI)	***p*** value
**List of donor- and cell-related variables predictive for osteoblastic differentiation (*R***^**2**^ **= 0.552)**			
Sex	47	20.83 (9.60; 32.05)	0.001
Osteoporosis	47	−27.38 (−45.65; −9.1	0.004
Vitamin D supplementation	47	27.08 (9.58; 44.58)	0.003
CD146+ cells (%)	47	0.28 (0.10; 0.46)	0.004
ALP+ cells (%)	47	0.51 (0.16; 0.87)	0.005
CD14+ cells (%)	47	−0.69 (−1.49; 0.11)	0.089
**List of donor- and cell-related variables predictive for adipocytic differentiation (*R***^**2**^ **= 0.557)**			
Sex	47	−7.95 (−16.24; 0.33)	0.060
Osteoporosis	47	17.36 (8.95; 25.76)	0.000
Alcohol consumption	47	−6.85 (−14.96; 1.26)	0.095
CD146+ cells (%)	47	−0.21 (−0.36; −0.05)	0.009
ALP+ cells (%)	47	0.31 (0.05; 0.57)	0.020
PDGFRα+ cells (%)	47	0.22 (0.04; 0.39)	0.016
CD362+ cells (%)	47	−0.17 (−0.36; 0.02)	0.073

Employing the same model, seven factors were selected as predictive for AD differentiation outcome. Four of them: sex of the donor (*p* = 0.006), presence of osteoporosis (*p* < 0.001), number of CD146+ cells (*p* = 0.009), and number of ALP+ cells (*p* = 0.02) are in common with the findings for OB differentiation but except for the ALP+ expression; these have the opposite effect on adipocyte formation. Three additional variables were predictive for AD differentiation: the number of PDGFRα+ cells (*p* = 0.016), CD362+ cells (*p* = 0.073), and alcohol consumption (*p* = 0.095). Combined, all predictive variables explain more than 50% of the variation observed within the data (*R*^2^ = 0.552 for OB differentiation and *R*^2^ = 0.557 for AD differentiation).

## Discussion

In the current study, we demonstrated that by combining key characteristics of donors and of cultured hBMSC strains, we were able to generate a model of collective variables that predicts > 50% of variation in osteoblast and adipocyte differentiation outcome. The model identified the following factors for selecting cells with the enhanced ability for in vitro mineralized matrix formation: male sex, absence of osteoporosis, current intake of vitamin D supplementation, and a higher number of ALP+ and CD146+ in cell cultures.

Both uni- and multivariable analysis revealed that the sex of the donor is a significant predictor for OB differentiation outcome. In contrast to our study, a number of previous reports did not detect a significant impact of donor sex on the number of committed OB or their differentiation outcome [23, 37–40]. For example, we were not able to detect sex differences of cultured hBMSC in one of our previous studies, which only included a cohort of younger donors, aged 20-35 [39]. One could presume that sex differences in the biology of hBMSCs became detectable with aging as we in the current study also included older patients. However, we could not demonstrate any significant interaction between sex and age on predicting OB and AD differentiation potency when applying multivariable analysis. Our data thus suggest that sex itself is an independent predictor for OB and AD differentiation outcome.

The influence of donor age on the biological properties of the hBMSCs has been previously examined in both animal and human studies [41], and the reported results have not been consistent. Some studies have reported that the hBMSCs obtained from elderly donors exhibit a reduced expression of osteoblastic gene markers, ALP activity, and decreased mineralized matrix formation [42–44], while others reported no age-related effects on the ability of the cells for OB or AD differentiation [23, 45, 46]. In vivo transplantation studies of hBMSCs have demonstrated similar bone-forming capacity regardless of donor age [25, 47, 48]. Interestingly, we have previously reported that the effect of donor age on hBMSC biology was only detectable during a long term (>60 days) in vitro culture and was caused by the accumulation of senescent cells [25], suggesting donor age effects may not be detectable in short-term cultures. In addition, the age-related effects were reported mainly on the proliferative capacity of hBMSCs, demonstrating an increased proliferative rate in cells from younger donors with the growing skeleton (<20 years) compared to the cells obtained from elderly donors (>60 years) [41]. Nevertheless, our study corroborates our previous report that hBMSCs obtained from the elderly do maintain their differentiation capacity and thus can be employed in autologous transplantation protocols [39].

In addition to sex and age, we examined the effect of a large number of clinical variables on OB and AD differentiation outcome. We observed that fracture age (<7 days) was positively associated with increased OB differentiation in male donors which may be related to the increased number of osteoblastic stem cells recruited for fracture repair or the release of bone enhancing growth factors [49]. We also observed that increased donor weight, but not BMI, was associated with enhanced AD differentiation outcome in female donors which corroborated our recent study showing that hBMSCs derived from obese healthy donors exhibit enhanced OB and AD differentiation compared to lean [50].

In our study, bone marrow collection site did not influence hBMSC differentiation outcome, which corroborates findings in earlier reports [40, 45]. In the multivariable analysis model, the absence of osteoporosis and intake of vitamin D supplementation were significant predictors for enhanced OB differentiation outcome. Based on a number of previous studies, the effect of osteoporosis, on in vitro mineralized matrix formation has not been consistent with some studies reporting no effect [51] or reduced OB differentiation [52–54] when comparing cultured hBMSCs isolated from osteoporotic donors and controls. We have also previously reported that hBMSC cultures established from osteoporotic patients exhibited similar proliferation and differentiation capacities as age-matched controls [39, 46]. The discrepancy may be explained by the fact that the donor population of the current study exhibited a more severe osteoporotic phenotype and osteoporotic fracture. hBMSCs express the vitamin D receptor, and active metabolites of vitamin D promote OB differentiation by upregulating osteogenic gene expression, increasing ALP activity and extracellular mineralized matrix deposition in vitro [55–57]. The observed effects of donor/patient vitamin D supplement intake on enhanced ability of cultured hBMSCs for OB differentiation in the current study may be caused by epigenetic changes, which have been observed for other cell types [58] and might include histone deacetylation, which was shown to rejuvenate osteoblastogenesis in hBMSCs from elderly individuals [59]. Thus, vitamin D intake may have positive effects on bone regeneration by targeting hBMSCs.

We further observed a number of cellular characteristics that were predictive for hBMSC differentiation outcome. We found a positive correlation between the number of ALP+ cells and both OB and AD differentiation. ALP is commonly employed marker for early stages of osteoblastic differentiation. However, the expression of ALP has also been detected in AD precursors that reside in the bone marrow [60] and an increased expression of ALP was found in hBMSCs committed to OB and AD fate but not in cells committed to chondrocytes [61]. On the other hand, ALP activity at both at baseline and following OB induction is a good predictor of the ability of cells to become mature bone-forming osteoblastic cells, which corroborate the role of ALP as important factor for bone mineralization [62]. CD146 was another surface marker that showed a positive correlation with OB differentiation outcome. CD146 has been demonstrated to define stem cell populations within hBMSC cultures [29, 32, 63] self-renewing capacity, clonogenicity, and formation of hematopoietic microenvironment in vivo [29, 63].

We also observed that hBMSC strains enriched in cells positive for ALP, PDGFRα, and negative for CD146 and CD362 exhibited enhanced AD differentiation. A similar observation has been reported by Uezumi et al., who demonstrated that human skeletal muscle progenitors enriched in PDGFRα+ cells possess high adipogenic differentiation potential [64]. On the other hand, Samsonraj et al. demonstrated that the high expression of PDGFRα is associated with high proliferation and increased ability to form bone tissue in vivo [65]. This discrepancy could be explained as PDGFRα is a marker of cells committed to both OB and AD. In our study, we observed a significant positive correlation between CD34 and AD differentiation. CD34 is commonly used as marker for hematopoietic stem cells, but a number of studies have reported that CD34 is also expressed in bone marrow stromal cells among cells with adipogenic [66] or osteoblastic differentiation potential [30].

Our study has some limitations. The hBMSCs were isolated from patients undergoing orthopedic surgery and thus may not represent a random sample of the whole population. Second, due to a limited cell number available from each donor, we examined the differentiation potential into osteoblasts and adipocytes but not to chondrocytes which is a relevant alternative lineage that informs about the “stemness” nature of the cultured cells. Third, we employed a standard 2D-culture system to test the differentiation potential of cultured hBMSCs; however, testing the cells in 3D-culture or testing their ability to form bone organoids may be relevant with respect to clinical applications especially the use of cells in bone tissue engineering as we have reported previously [67]. Finally, the described multivariable model was able to explain only 55% of the observed variations of the osteoblast differentiation outcome, suggesting the presence of smaller effects of multiple additional variables not detected due to the power of the current study and the results need to be validated in independent cohorts.

## Conclusions

Our study provides a number of clinically relevant variables that can be tested in prospective clinical trials or in preclinical animal models for bone tissue regeneration. Our findings indicate that the hBMSCs collected from non-osteoporotic male donors with vitamin D supplementation and enriched in fraction of CD146+/ALP+/CD14- cells have enhanced OB differentiation potency; by more than 50%. Moreover, our analysis demonstrates that the hBMSCs collected from osteoporotic females, enriched in subpopulations of ALP+/PDGFRα+/CD146-/CD362- cells, had more than 50% of probability to exhibit an enhanced differentiation potency toward adipocytes formation; considered a non-desired differentiation outcome. Importantly, we think that the variables proposed here are relevant for choosing the most suitable donors to obtain hBMSCs with optimal osteoblast differentiation capacity, potentially improving the clinical therapeutic outcome of hBMSC-based bone regeneration.

## Supplementary Information

**Additional file 1: Supplementary Figure 1.** Comparison of osteoblastic differentiation outcome measures at day 14 of osteoblastic differentiation. Graphs illustrate the different parameters which in addition to (A) alizarin red could serve as osteoblastic differentiation outcome measures, including (B) *BSP* gene expression, (C) *COL1* gene expression, (D) *OCN* gene expression. The values shown are analyzed from 10 samples with low osteoblastic differentiation potency (low OB) and 10 samples with high osteoblastic differentiation potency (high OB). Data were analyzed using unpaired Mann-Whitney test with the significance of *p* < 0.05.

**Additional file 2: Supplementary Figure 2.** Correlation between gene expression and osteoblastic differentiation outcome. Graphs illustrate the correlations between (A) *BSP*, (B) *COL1*, (C) *OCN* gene expression at day 14 after osteoblastic induction and osteoblastic differentiation outcome. (D) Relationship between combined (multiplied) expression of *BSP*, *COL1* and *OCN* genes expressed in arbitrary units (AU) and osteoblastic differentiation potency. Correlations were analyzed using Spearman correlation test. Each dot represents the average value of cultured cells from a single donor, rs = Spearman's rank correlation coefficient and n = number of tested cell strains, each derived from a single donor.

**Additional file 3: Supplementary Figure 3.** Correlation between cell proliferation and osteoblast and adipocyte differentiation outcome of hBMSCs. Graphs illustrate the correlation between cell proliferation and (A) osteoblastic and (B) adipocytic differentiation outcome. Correlations were analyzed using Spearman correlation test. Each dot represents the average value of cultured cells from a single donor, r_s_ = Spearman's rank correlation coefficient and n = number of tested cell strains, each derived from a single donor.

**Additional file 4: Supplementary Figure 4.** Correlation of colony forming unit-fibroblast (CFU-f) and differentiation outcome of hBMSCs. (A) Microphotographs illustrate formation of CFU-f in hBMSC cultures. The ALP positive colonies were stained and marked (black dots) prior to visualization of total number of colonies using crystal violet. (B, D) The correlation of total colony number, (C, E) number of ALP positive colonies and osteoblastic and adipocytic differentiation outcome. Correlations were analyzed using Spearman correlation test. Each dot represents the average value of cultured cells from a single donor, r_s_ = Spearman's rank correlation coefficient and n = number of tested cell strains, each derived from a single donor.

**Additional file 5: Supplementary Figure 5.** Correlation between expression of cell surface markers and differentiation outcome of hBMSCs. The relationship between osteoblastic and adipocytic differentiation outcome of hBMSCs and percentage of (A, B) CD271+, (C, D) PDPN+, (E, F) CD164+, (G, H) CD362+ and (I, J) CXCR4+ cells. The relationships were analyzed using Spearman correlation test. Each dot represents the average value of cultured cells from a single donor, r_s_ = Spearman's rank correlation coefficient and n = number of tested cell strains, each derived from a single donor.

**Additional file 6: Supplementary Table 1.** List of genes and primer sequences used for qRT-PCR.

**Additional file 7: Supplementary Table 2.** A list of correlation coefficients of donor characteristics and osteoblastic or adipocytic differentiation outcome of cultured human bone marrow stromal cells (hBMSCs).

**Additional file 8: Supplementary Table 3.** The effect of donor characteristic variables on the osteoblastic and adipocytic differentiation outcome of cultured human bone marrow stromal cells (hBMSCs).

**Additional file 9: Supplementary Table 4.** Correlation between cellular characteristics and differentiation potency of hBMSCs.

## Data Availability

The data that support the findings of this study are available on reasonable request from the corresponding author. The data are not publicly available due to privacy or ethical restrictions.

## Abbreviations

AD: Adipocytic differentiation
ALP: Alkaline phosphatase
AUC: Area under the curve
BMI: Body mass index
BRL: Rosiglitazone
*BSP*: Bone sialoprotein
CFU-f: Colony-forming unit-fibroblast
*COL1A*: Collagen 1
DMEM: Dulbecco’s modified Eagle’s medium
EDTA: Ethylenediaminetetraacetic acid
FBS: Fetal bovine serum
hBMSCs: Human bone marrow stromal cells
IBMX: 3-Isobutyl-1-methylxanthine
MEM: Minimum essential medium
OB: Osteoblastic cells
*OCN*: Osteocalcin
PBS: Phosphate-buffered saline
PDGFRα: Platelet-derived growth factor receptor A
PDPN: Podoplanin
PDT: Population doubling time
PFA: Paraformaldehyde
P/S: Penicillin/streptomycin
qRT-PCR: Quantitative real-time PCR
