# Supplementary figures and images for: Identification of a clinical signature predictive of differentiation fate of human bone marrow stromal cells
Source: Stem Cell Res Ther. 2021 May 3;12:265. doi: 10.1186/s13287-021-02338-1 (PMC8091554; doi:10.1186/s13287-021-02338-1)

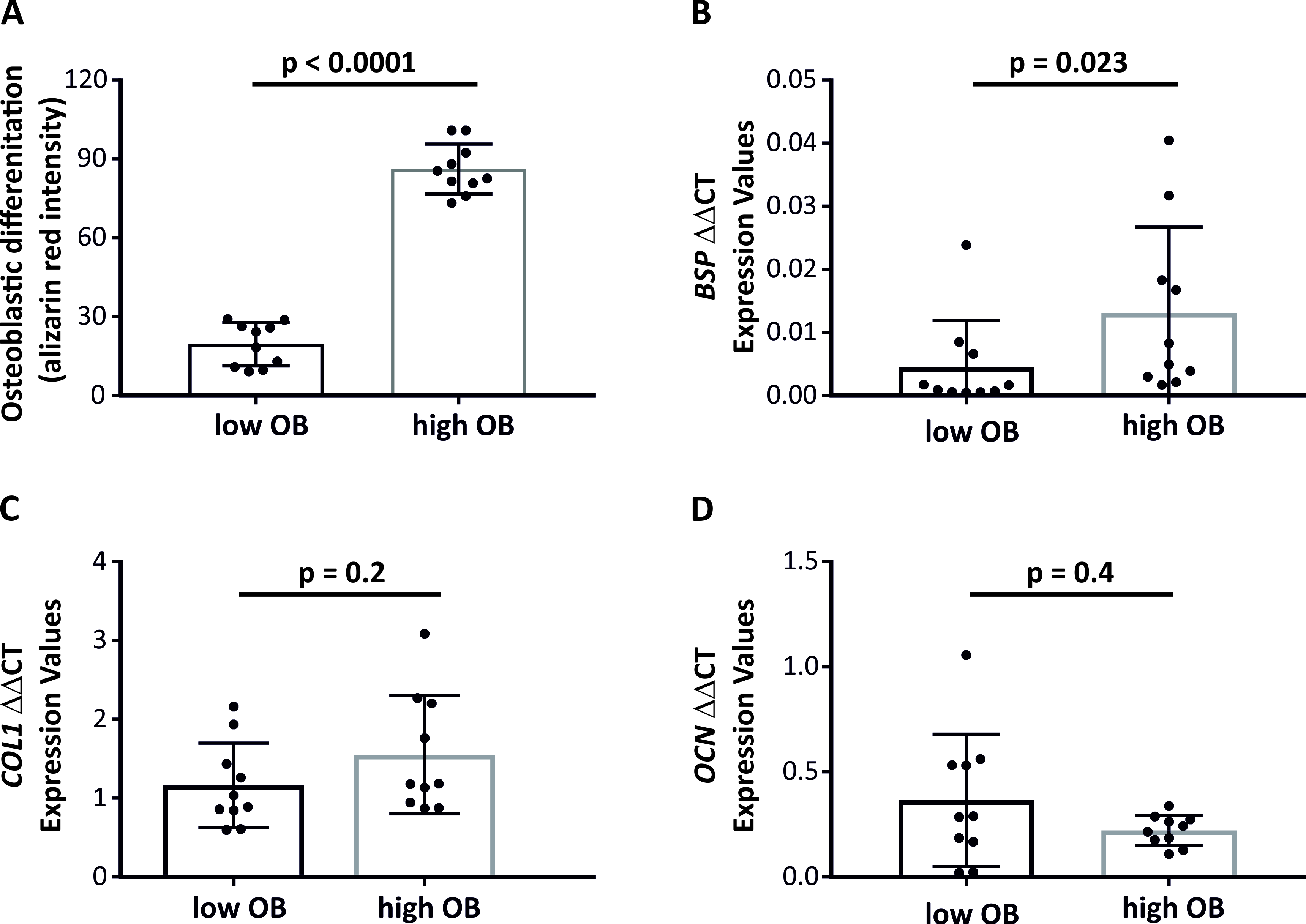

Supplement: Supplementary file 1 — Additional file 1: Supplementary Figure 1. Comparison of osteoblastic differentiation outcome measures at day 14 of osteoblastic differentiation. Graphs illustrate the different parameters which in addition to (A) alizarin red could serve as osteoblastic differentiation outcome measures, including (B) BSP gene expression, (C) COL1 gene expression, (D) OCN gene expression. The values shown are analyzed from 10 samples with low osteoblastic differentiation potency (low OB) and 10 samples with high osteoblastic differentiation potency (high OB). Data were analyzed using unpaired Mann-Whitney test with the significance of p < 0.05. [file 13287_2021_2338_MOESM1_ESM.tif]

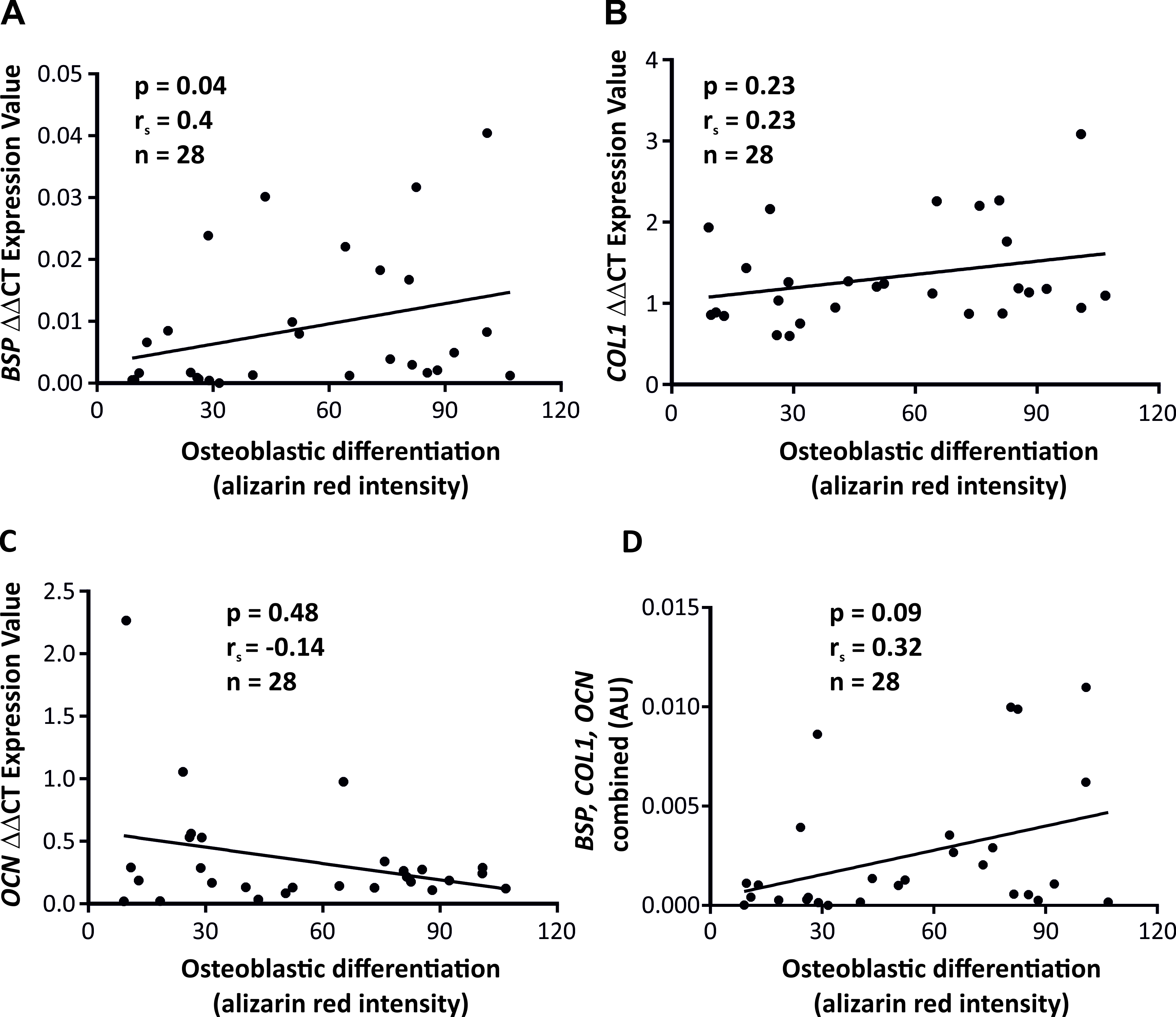

Supplement: Supplementary file 2 — Additional file 2: Supplementary Figure 2. Correlation between gene expression and osteoblastic differentiation outcome. Graphs illustrate the correlations between (A) BSP, (B) COL1, (C) OCN gene expression at day 14 after osteoblastic induction and osteoblastic differentiation outcome. (D) Relationship between combined (multiplied) expression of BSP, COL1 and OCN genes expressed in arbitrary units (AU) and osteoblastic differentiation potency. Correlations were analyzed using Spearman correlation test. Each dot represents the average value of cultured cells from a single donor, rs = Spearman's rank correlation coefficient and n = number of tested cell strains, each derived from a single donor. [file 13287_2021_2338_MOESM2_ESM.tif]

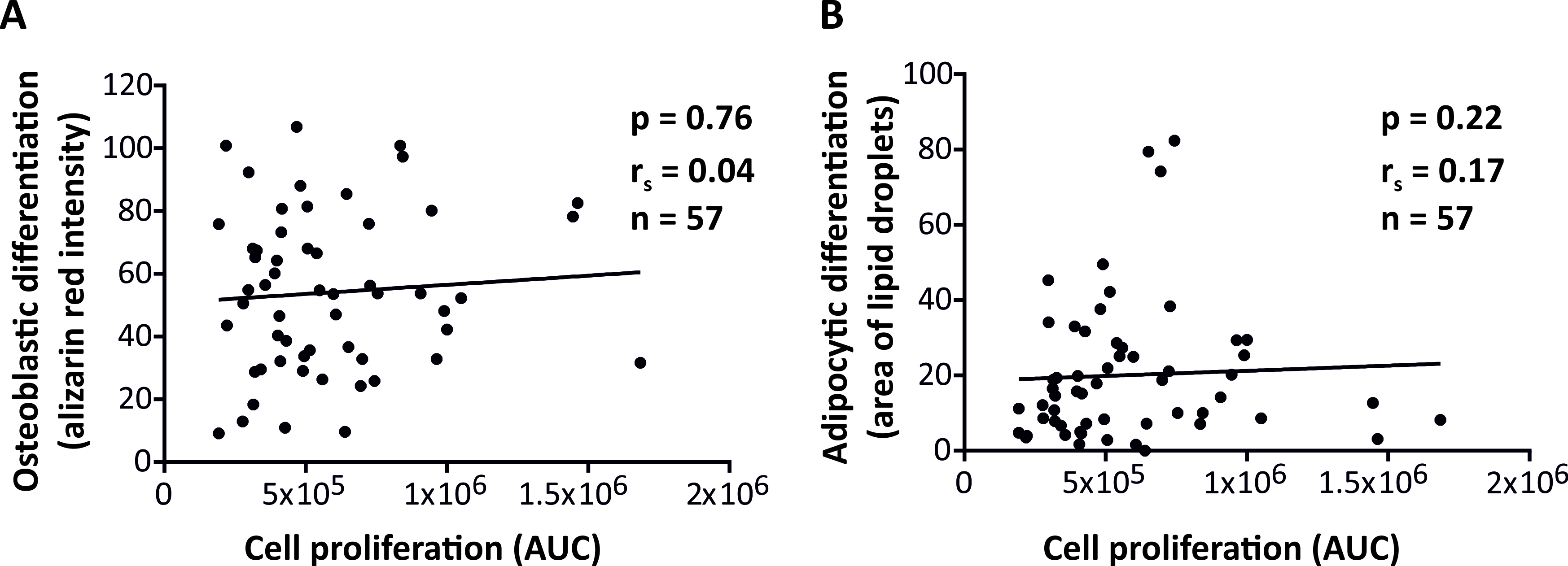

Supplement: Supplementary file 3 — Additional file 3: Supplementary Figure 3. Correlation between cell proliferation and osteoblast and adipocyte differentiation outcome of hBMSCs. Graphs illustrate the correlation between cell proliferation and (A) osteoblastic and (B) adipocytic differentiation outcome. Correlations were analyzed using Spearman correlation test. Each dot represents the average value of cultured cells from a single donor, rs = Spearman's rank correlation coefficient and n = number of tested cell strains, each derived from a single donor. [file 13287_2021_2338_MOESM3_ESM.tif]

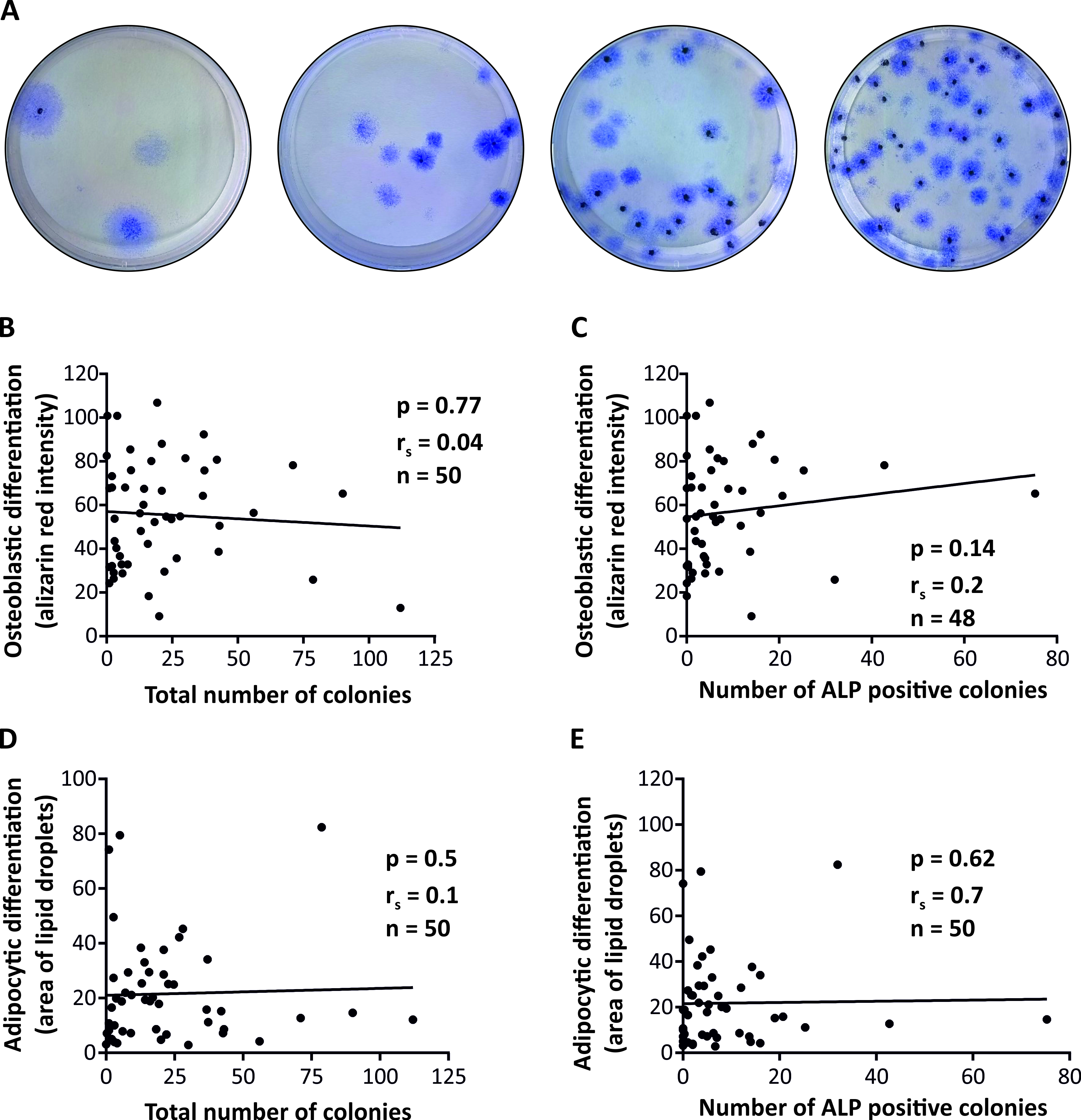

Supplement: Supplementary file 4 — Additional file 4: Supplementary Figure 4. Correlation of colony forming unit-fibroblast (CFU-f) and differentiation outcome of hBMSCs. (A) Microphotographs illustrate formation of CFU-f in hBMSC cultures. The ALP positive colonies were stained and marked (black dots) prior to visualization of total number of colonies using crystal violet. (B, D) The correlation of total colony number, (C, E) number of ALP positive colonies and osteoblastic and adipocytic differentiation outcome. Correlations were analyzed using Spearman correlation test. Each dot represents the average value of cultured cells from a single donor, rs = Spearman's rank correlation coefficient and n = number of tested cell strains, each derived from a single donor. [file 13287_2021_2338_MOESM4_ESM.tif]

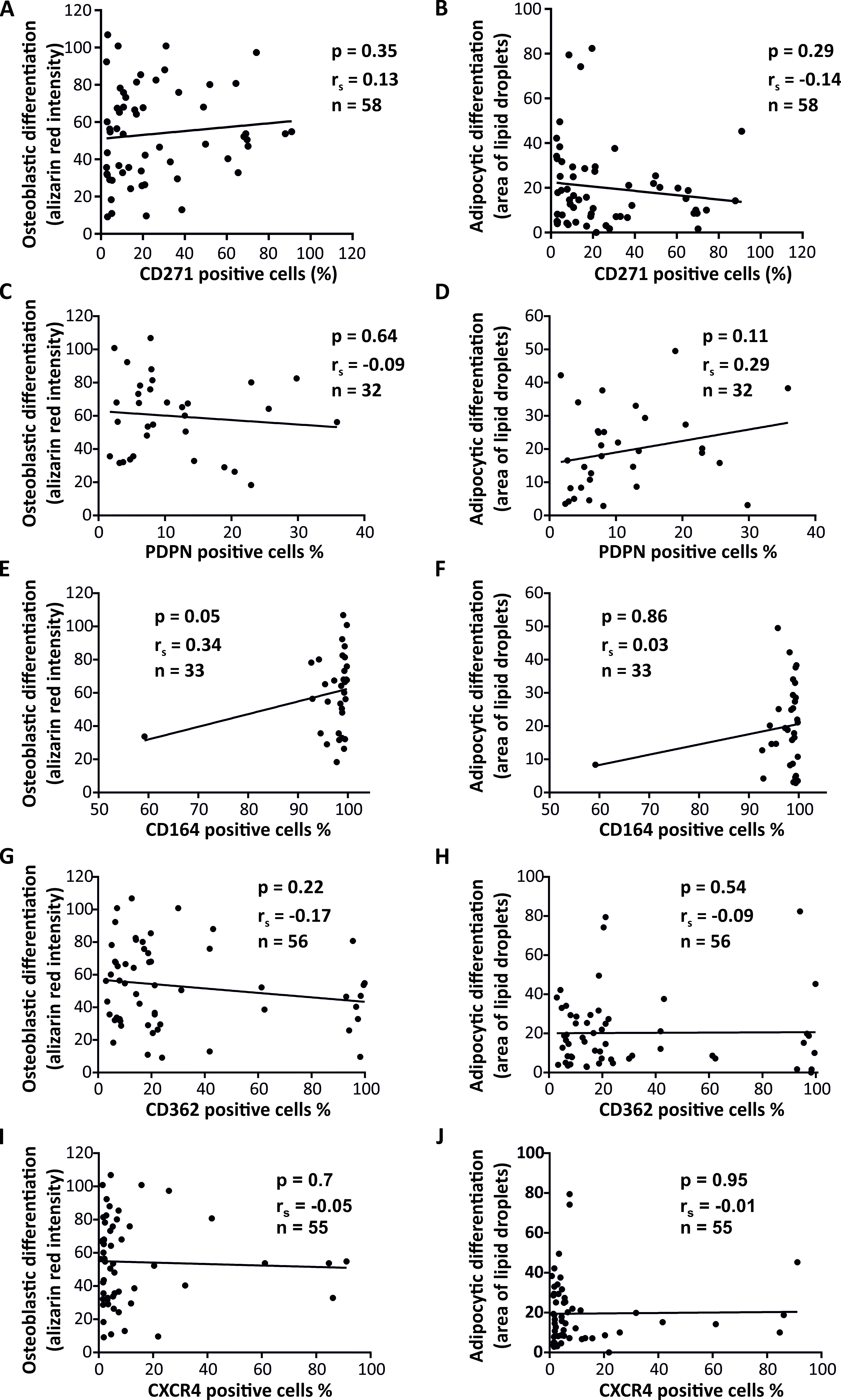

Supplement: Supplementary file 5 — Additional file 5: Supplementary Figure 5. Correlation between expression of cell surface markers and differentiation outcome of hBMSCs. The relationship between osteoblastic and adipocytic differentiation outcome of hBMSCs and percentage of (A, B) CD271+, (C, D) PDPN+, (E, F) CD164+, (G, H) CD362+ and (I, J) CXCR4+ cells. The relationships were analyzed using Spearman correlation test. Each dot represents the average value of cultured cells from a single donor, rs = Spearman's rank correlation coefficient and n = number of tested cell strains, each derived from a single donor. [file 13287_2021_2338_MOESM5_ESM.tif]
