# Supplementary material for: Identification of a clinical signature predictive of differentiation fate of human bone marrow stromal cells
Source: Stem Cell Res Ther. 2021 May 3;12:265. doi: 10.1186/s13287-021-02338-1 (PMC8091554; doi:10.1186/s13287-021-02338-1)
Supplement: Supplementary file 6 — Additional file 6: Supplementary Table 1. List of genes and primer sequences used for qRT-PCR. [file 13287_2021_2338_MOESM6_ESM.docx]

**Supplementary Table 1**

**List of genes and primer sequences used for qRT-PCR.**

| **Gene** | **Forward sequence** | **Reverse sequence** |
| --- | --- | --- |
| BSP^#^ | GGGCTATGGAGAGGACGCCACGCCT | TCCCCAGCCTTCTTGGGAAGCTGGATT |
| COL1A1^[1]^ | AGGGCTCCAACGAGATCGAGATCCG | TACAGGAAGCAGACAGGGCCAACGTCG |
| OCN ^[1]^ | CATGAGAGCCCTCACA | AGAGCGACACCCTAGAC |
| β-actin ^[2]^ | ATTGGCAATGAGCGGTTCCG | AGGGCAGTGATCTCCTTCTG |

The primers used in the current study are both, self-designed (# - self-designed primers) or taken from the previous studies published by our research group and referred to the particular articles.

1. Twine NA, Harkness L, Kassem M et al. Transcription factor ZNF25 is associated with osteoblast differentiation of human skeletal stem cells. BMC Genomics. 2016;17:872.

2. Chen L, Shi K, Andersen TL et al. KIAA1199 is a secreted molecule that enhances osteoblastic stem cell migration and recruitment. Cell Death Dis. 2019;10:126.
