# Supplementary material for: Identification of a clinical signature predictive of differentiation fate of human bone marrow stromal cells
Source: Stem Cell Res Ther. 2021 May 3;12:265. doi: 10.1186/s13287-021-02338-1 (PMC8091554; doi:10.1186/s13287-021-02338-1)
Supplement: Supplementary file 7 — Additional file 7: Supplementary Table 2. A list of correlation coefficients of donor characteristics and osteoblastic or adipocytic differentiation outcome of cultured human bone marrow stromal cells (hBMSCs). [file 13287_2021_2338_MOESM7_ESM.docx]

**Supplementary Table 2**

**A list of correlation coefficients of donor characteristics and osteoblastic or adipocytic differentiation outcome of cultured human bone marrow stromal cells (hBMSCs).**

| **Numerical variables** | **Donors** | **N of subjects** | **Osteoblastic differentiation (alizarin red intensity)** | **Adipocytic differentiation (area of lipid droplets)** |
| --- | --- | --- | --- | --- |
|  |  |  |  |  |
|  |  |  | **p value** | **p value** |
| Age | Females | 32 | 0.29 | 0.53 |
|  | Males | 26 | 0.66 | 0.89 |
| Weight | Females | 32 | 0.69 | **0.03** |
|  | Males | 26 | **0.06** | 0.9 |
|  | Total | 58 | 0.66 | 0.92 |
| Height | Females | 32 | 0.71 | 0.15 |
|  | Males | 26 | 0.36 | 0.76 |
|  | Total | 58 | 0.38 | 0.88 |
| BMI | Females | 32 | 0.77 | 0.26 |
|  | Males | 26 | 0.12 | 0.83 |
| Haemoglobin | Female | 28 | 0.43 | 0.93 |
|  | Male | 21 | 0.83 | 0.44 |
|  | Total | 49 | 0.74 | 0.5 |
| Leucocytes | Females | 26 | 0.61 | 0.74 |
|  | Males | 22 | 0.76 | 0.78 |
|  | Total | 48 | 0.92 | 0.1 |
| CRP | Females | 27 | 0.46 | 0.75 |
|  | Males | 22 | 0.41 | 0.11 |
|  | Total | 49 | 0.67 | 0.39 |

Note - Osteoblastic differentiation outcome was assessed by formation of extracellular mineralised matrix by hBMSCs and quantified by intensity of alizarin red staining expressed in arbitrary units (AU). Adipocytic differentiation outcome was measured as potency of hBMSCs to from adipocytes and quantified as area of lipid droplets (expressed in arbitrary units, AU) visualised by oil red o staining. The univariable analyses were performed using Pearson (for variables that showed normal distribution) or Spearman two-tailed correlation test (for variables that did not show normal distribution. Red: p<0.05, orange: p<0.1
