# Supplementary material for: Identification of a clinical signature predictive of differentiation fate of human bone marrow stromal cells
Source: Stem Cell Res Ther. 2021 May 3;12:265. doi: 10.1186/s13287-021-02338-1 (PMC8091554; doi:10.1186/s13287-021-02338-1)
Supplement: Supplementary file 8 — Additional file 8: Supplementary Table 3. The effect of donor characteristic variables on the osteoblastic and adipocytic differentiation outcome of cultured human bone marrow stromal cells (hBMSCs). [file 13287_2021_2338_MOESM8_ESM.docx]

**Supplementary Table 3**

**The effect of donor characteristic variables on the osteoblastic and adipocytic differentiation outcome of cultured human bone marrow stromal cells (hBMSCs).**

| **Variable** | **Donors** | **N of subjects** | **Osteoblastic differentiation**  **(alizarin red intensity)** | | **Adipocytic differentiation**  **(area of lipid droplets)** | |
| --- | --- | --- | --- | --- | --- | --- |
|  |  |  |  |  |  |  |
|  |  |  | **Mean ± SD** | **p value** | **Mean ± SD** | **p value** |
| Subjects with and without osteoporosis | Females | 17 vs 15 | (50.8 ± 25.3) vs (42.9 ± 16.1) | 0.31 | (16.3 ± 19.2) vs (27.7 ± 24.3) | 0.1 |
|  | Males | 5 vs 21 | (60.2 ± 19) vs (62.3 ± 29) | 0.80 | (18.1 ± 15.8) vs (18 ± 11.5) | 0.95 |
|  | Total | 22 vs 36 | (52.9 ± 24) vs (54.2 ± 26.1) | 0.86 | (16.7 ± 18.2) vs (22 ± 18.3) | 0.15 |
| Subjects with and without hypertension | Females | 11 vs 21 | (42.52 ± 23.7) vs (49.47 ± 20.6) | 0.40 | (12.1 ± 7.7) vs (26.7 ± 25.6) | **0.08** |
|  | Males | 10 vs 16 | (59.6 ± 26.3) vs (63.4 ± 28.4) | 0.73 | (19.2 ± 13.1) vs (17.2 ± 11.7) | 0.69 |
|  | Total | 21 vs 37 | (50.6 ± 25.8) vs (55.5 ± 25) | 0.48 | (15.5 ± 11) vs (22.6 ± 21) | 0.39 |
| Subjects with and without type 2 diabetes | Females | 2 donors only | NA | NA | NA | NA |
|  | Males | 8 vs 18 | (57 ± 22) vs (64.1 ± 29.5) | 0.54 | (20.4 ± 13) vs (16.9 ± 22.7) | 0.5 |
|  | Total | 10 vs 48 | (55.5 ± 20.9) vs (53.4 ± 26.1) | 0.81 | (18.6 ± 12.2) vs (20.3 ± 19.4) | 0.71 |
| Subjects taking less and more than 3 medicines | Females | 26 vs 6 | (44.4 ± 19.2) vs (58.5 ± 28.9) | 0.17 | (23 ± 24.3) vs (15.8 ± 6.3) | 0.91 |
|  | Males | 19 vs 7 | (62.8 ± 27.2) vs (59.6 ± 29) | 0.61 | (18.8 ± 12.5) vs (15.7 ± 11.3) | 0.57 |
|  | Total | 45 vs 13 | (52.2 ± 24.4) vs (59.1 ± 27.8) | 0.39 | (21.2 ± 20.1) vs (15.7 ± 8.9) | 0.76 |
| Subjects with and without morphine treatment | Females | 20 vs 12 | (47.64 ± 5.53) vs (46.15 ± 4.59) | 0.85 | (21.9 ± 22) vs (21.1 ± 23.3) | 0.63 |
|  | Males | 18 vs 8 | (56.1 ± 28.7) vs (74.9 ± 18.8) | 0.11 | (16.8 ± 12.3) vs (20.7 ± 11.6) | 0.45 |
|  | Total | 38 vs 20 | (51.7 ± 26.6) vs (57.6 ± 22) | 0.39 | (19.5 ± 18) vs (20.1 ± 19.1) | 0.79 |
| Subjects with and without paracetamol treatment | Females | 27 vs 5 | (49 ± 22.8) vs (36,7 ± 7.4) | 0.26 | (23.7 ± 23.2) vs (10.5 ± 10.9) | 0.18 |
|  | Males | 2 donors without treatment | NA | NA | NA | NA |
|  | Total | 51 vs 7 | (55.3 ± 25.9) vs (42.6 ± 15.9) | 0.18 | (21.4 ± 18.8) vs (10.1 ± 9) | **0.08** |
| Subjects with and without pantoprasol treatment | Females | 5 vs 27 | (66.7 ± 21.9) vs (43.6 ± 20) | **0.06** | (13.7 ± 9.4) vs (23.1 ± 23.6) | 0.61 |
|  | Males | only 1 donor taking pantoprasol | NA | NA | NA | NA |
|  | Total | 6 vs 52 | (65.8 ± 19.6) vs (52.3 ± 25. 5) | 0.24 | (16.2 ± 10.4) vs (20.4 ± 19) | 0.85 |
| Subjects with and without statins treatment | Females | 3 vs 29 | NA | NA | NA | NA |
|  | Males | 6 vs 20 | (73.2 ± 19.6) vs (58.5 ± 28.6) | 0.39 | (14.8 ± 7.9) vs (19 ± 13) | 0.66 |
|  | Total | 9 vs 49 | (61 ± 2,.8) vs (52.4 ± 24.7) | 0.35 | (13.8 ± 6.9) vs (21.1 ± 19.5) | 0.52 |
| Subjects with and without alendronate treatment | Females | 5 vs 27 | (61.9 ± 25.1) vs (44.3 ± 20.1) | 0.10 | (15.8 ± 13.1) vs (22.7 ± 23.5) | 0.88 |
|  | Males | none | NA | NA | NA | NA |
|  | Total | 5 vs 53 | (61.9 ± 25.1) vs (53 ± 25.2) | 0.43 | (15.8 ± 13.1) vs (20.4 ± 18.7) | 0.73 |
| Subjects with and without vitamin D supplementation | Females | 13 vs 19 | (45.9 ± 19.4) vs (47.9 ± 28.7) | 0.81 | (17.8 ± 21.8) vs (24.3 ± 22.6) | 0.25 |
|  | Males | 7 vs 19 | (56.1 ± 23.3) vs (64 ± 28.6) | 0.50 | (16.3 ± 13.9) vs (18.6 ± 11.6) | 0.65 |
|  | Total | 20 vs 38 | (49.5 ± 20.8) vs (56 ± 27.1) | 0.36 | (17.3 ± 19) vs (21.4 ± 18) | 0.21 |
| Subjects with and without calcium supplementation | Females | 13 vs 19 | (45.9 ± 19.4) vs (47.9 ± 23.4) | 0.81 | (17.8 ± 21.8) vs (24.3 ± 22.6) | 0.25 |
|  | Males | 7 vs 19 | (59.5 ± 24.3) vs (62.8 ± 28.7) | 0.91 | (18 ± 13.5) vs (18 ± 11.8) | 0.96 |
|  | Total | 20 vs 38 | (50.7 ± 21.7) vs (55.3 ± 27) | 0.51 | (17.9 ± 19) vs (21.1 ± 18.1) | 0.35 |
| Fracture age (less and more than 7 days) | Females | 18 vs 11 | (47.5 ± 24) vs (44.3 ± 17) | 0.70 | (23 ± 24.2) vs (21.2 ± 22) | 0.98 |
|  | Males | 15 vs 9 | (70.4 ± 22.2) vs (45.9 ± 31.5) | **0.04** | (17.6 ± 11.8) vs (18,5 ± 14.4) | 0.86 |
|  | Total | 33 vs 20 | (57.9 ± 25.6) vs (45 ± 23.9) | 0.07 | (20.5 ± 19,5) vs (20 ± 18.5) | 0.97 |
| Subjects currently smoking cigarettes (yes vs no) | Females | 9 vs 23 | (56.5 ± 22.8) vs (43.4 ± 20.4) | 0.12 | (20 ± 24.2) vs (22.3 ± 21.9) | 0.87 |
|  | Males | 12 vs 14 | (61.3 ± 23) vs (62.4 ± 31.1) | 0.92 | (17.7 ± 11.9) vs (18.2 ± 12.6) | 0.93 |
|  | Total | 21 vs 37 | (59.2 ± 22.5) vs (50.6 ± 26.3) | 0.21 | (18.7 ± 17.7) vs (20.7 ± 18.8) | 0.88 |
| Subjects consuming alcohol (yes vs no) | Females | 18 vs 14 | (47.6 ± 16.6) vs (46.4 ± 27.3) | 0.89 | (23,7 ± 24.4) vs (19 ± 19.5) | 0.64 |
|  | Males | 20 vs 6 | (62.8 ± 28.9) vs (59 ± 22.4) | 0.79 | (18.3 ± 12.7) vs (17 ± 10.3) | 0.88 |
|  | Total | 38 vs 20 | (55.6 ± 24.8) vs (50 ± 26) | 0.44 | (20.8 ± 19.1) vs (18.4 ± 17) | 0.66 |
| CRP (less and more than 10) | Females | 19 vs 8 | (51 ± 23.7) vs (45.5 ± 20.9) | 0.58 | (20.4 ± 22.4) vs (18.5 ± 14) | 0.77 |
|  | Males | 6 vs 16 | (60.2 ± 18.8) vs (63.2 ± 28.2) | 0.75 | (17.9 ± 7.21) vs (16.5 ± 13.5) | 0.64 |
|  | Total | 25 vs 24 | (53.2 ± 22.6) vs (57.3 ± 27.9) | 0.56 | (19.8 ± 19.7) vs (17.2 ± 13.4) | 0.83 |

Note - Osteoblastic differentiation outcome was assessed by formation of extracellular mineralised matrix by hBMSCs and quantified by intensity of alizarin red staining expressed in arbitrary units (AU). Adipocytic differentiation outcome was measured as potency of hBMSCs to from adipocytes and quantified as area of lipid droplets (expressed in arbitrary units, AU) visualised by oil red o staining. The univariable analysis between groups were performed using Student-t test (for variables that were normally distributed) or Mann-Whitney test (for variables that did not follow normal distribution). The data are presented as mean values ± SD. NA- non applicable, red: p<0.05, orange: p<0.1
