# Supplementary material for: Identification of a clinical signature predictive of differentiation fate of human bone marrow stromal cells
Source: Stem Cell Res Ther. 2021 May 3;12:265. doi: 10.1186/s13287-021-02338-1 (PMC8091554; doi:10.1186/s13287-021-02338-1)
Supplement: Supplementary file 9 — Additional file 9: Supplementary Table 4. Correlation between cellular characteristics and differentiation potency of hBMSCs. [file 13287_2021_2338_MOESM9_ESM.docx]

**Supplementary Table 4**

**Correlation between cellular characteristics and differentiation potency of hBMSCs.**

| **Variable** | **Donors** | **N of subjects** | **p value** | |
| --- | --- | --- | --- | --- |
|  |  |  | **OB** | **AD** |
| Cell proliferation (AUC) | Females | 32 | 0.42 | **0.07** |
|  | Males | 25 | 0.53 | 0.58 |
| Number of ALP positive colonies per 1 million seeded cells | Females | 26 | 0.29 | 0.65 |
|  | Males | 22 | 0.42 | 0.21 |
| Total number of colonies per 1 million seeded cells | Females | 28 | 0.68 | 0.85 |
|  | Males | 22 | 0.71 | 0.24 |

Note - Human bone marrow stromal cells (hBMSCs) were obtained from 58 donors undergoing surgery for bone fractures and were induced into osteoblasts or adipocytes. Osteoblastic differentiation outcome (OB) was assessed by formation of extracellular mineralised matrix by hBMSCs and quantified by intensity of alizarin red staining expressed in arbitrary units (AU). Adipocytic differentiation outcome (AD) was measured as potency of hBMSCs to from adipocytes and quantified as area of lipid droplets (expressed in arbitrary units, AU) visualised by oil red o staining. The univariable analyses were performed using Pearson (for variables that showed normal distribution) or Spearman two-tailed correlation test (for variables that did not have a normal distribution). Orange: p<0.1
